# Multiple myeloma and physical activity

**DOI:** 10.1186/s13104-021-05591-y

**Published:** 2021-05-07

**Authors:** Catherine S. Y. Lecat, Orla McCourt, Joanne Land, Kwee Yong, Abigail Fisher

**Affiliations:** 1 University College London Cancer Institute, 72 Huntley Street, London, WC1E 6DD UK; 2 University College London Hospitals NHS Foundation Trust, 235 Euston Road, London, NW1 2BU UK; 3 University College London Behavioural Science and Health, Institute of Epidemiology & Health, 1-19 Torrington Place, London, WC1E 6BT UK

**Keywords:** Multiple myeloma, Physical activity, Exercise

## Abstract

**Objective:**

Physical activity has been shown to improve quality of life in cancer patients with some evidence in multiple myeloma. This study aimed to determine myeloma patients’ exercise levels, their perception of physical activity, and to explore correlations with quality of life. Myeloma outpatients were invited to complete a number of questionnaires, including the Godin leisure-time exercise questionnaire (GLTEQ) to determine their exercise levels, the Functional Assessment of Cancer Therapy-General (FACT-G) questionnaire to assess health related quality of life, and the Functional Assessment of Chronic Illness Therapy-Fatigue (FACIT-F) questionnaire to assess fatigue.

**Results:**

Of the 65 respondents, 75% would like to increase their exercise level. Weakness, fatigue and pain were the most commonly perceived barriers to physical activity. 59% would like to receive physical activity advice. Only 25% were deemed active based on their GLTEQ scores. Finally, there was a significant positive correlation between the GLTEQ score and the FACT-G score (p < 0.001). Results highlight an unmet exercise need in myeloma patients. Current practice should be reviewed to develop a more holistic care model that incorporates tailored exercise advice or programme.

**Supplementary Information:**

The online version contains supplementary material available at 10.1186/s13104-021-05591-y.

## Introduction

Multiple myeloma (MM) is an incurable bone marrow malignancy with multiple relapses and remission periods, followed by eventual treatment resistant disease. Recent novel therapies have dramatically improved patients’ survival. MM survivors now face the challenge of regaining premorbid psychosocial and physical wellbeing, whilst coping with disease symptoms and treatment side effects. With osteolytic bone destruction occurring in nearly 90% of patients [1], physical activity (PA) is often perceived as increasing risk of injury and is not actively promoted. The lack of PA, along with long term toxicity from complex treatment regimens and autologous stem cell transplantation (ASCT), can lead to muscle atrophy, fatigue and de-functioning, thus negatively impact on quality of life (QoL) [2].

PA has been shown to associate with less fatigue, improved QoL and physical functioning in cancer patients [3–5]. Previous studies confirmed that tailored exercise programme in MM patients was safe and feasible, with high adherence, and was associated with benefits in QoL, fatigue and muscle strength [6, 7]. Additional studies are required to further confirm these benefits. Moreover, a better understanding of the prevailing attitudes toward PA in patients and clinical teams is needed if such programmes are to become part of survivorship care, and to be supported by healthcare professionals (HCP).

PA is recognised as an important aspect to the quality of cancer patients’ survival. The U.K. Independent Cancer Taskforce recommended that all cancer patients should receive tailored PA advice. In MM, such an initiative remains hampered by a lack of understanding and awareness of the benefits of PA, and how to promote safe exercise in the context of myeloma bone disease. We sought to find out MM patients’ attitude towards PA and ascertain their levels of PA and QoL in order to better understand their survivorship needs.

## Main text

### Methods

This is a service evaluation study conducted in the University College London Hospital, a tertiary myeloma centre. Patients attending MM outpatient clinics were invited to complete a survey (see Additional file 1), collecting demographic data and information on their MM status and treatment. PA questions include whether they would like to increase their exercise level, their perceived barrier(s) to PA, whether they would like to receive PA advice and their preferred way(s) to receive it.

PA level was assessed using the Godin leisure-time exercise questionnaire (GLTEQ) [8, 9]. A score of > 24 represents physically active, 14–23 represents moderately active, and < 14 represents insufficiently active. Functional Assessment of Cancer Therapy-General (FACT-G) and Functional Assessment of Chronic Illness Therapy-Fatigue (FACIT-F) questionnaires were used to assess QoL and fatigue respectively [10, 11].

Completion of this survey was optional and implied consent was obtained when patients returned the completed survey. All responses were anonymised. NHS Health Research Authority (HRA) ethics approval was not required based on the HRA decision tool [12] and the need for written consent was waived by HRA Research Ethics Committee (REC). Data were summarised descriptively and quantitative parameters were presented as percentages, medians, means and quartiles. Non-parametric Mann–Whitney U test and Kruskal–Wallis test were used to compare between groups. Associations between variables were analysed using the Spearman’s correlation test. A p-value of < 0.05 was deemed statistically significant. SPSS was used for statistical analyses and to generate figures.

### Results

Between March and May 2019, 65 patients completed the survey. Patient characteristics are shown in Table 1. Two-third (66%) were on myeloma treatment, with 26% on three or more lines. Median time from MM diagnosis was 3.8 years (range 0.1–15). 12% had been diagnosed for over 10 years. 75% would like to increase their PA level and 9% would not. The rest were either neutral or did not specify. The most commonly perceived barrier to improving PA was weakness (43%), fatigue (40%) and pain (26%). Other barriers include neuropathy from myeloma treatment (20%), myeloma bone disease (15%), arthritis (9%) and previous surgery limiting mobility (8%). Parkinsonism and previous nerve injury were stated for two respondents. 71% reported that a combination of factors, rather than a single factor, prevented them from improving PA (Table 2).

**Table 1:** Patient characteristics with the median, lower and upper quartiles of their GLTEQ, FACT-G and FACIT-F scores

Patient characteristics (n = 65)	Frequency (%)	GLTEQ score	p-value	FACT-G score	p-value	FACIT-F score	p-value
Gender							
Male	38 (58)	15 (3.8–31)	0.92	70.7 (58.1–94.1)	0.56	31 (16.8–40.3)	0.99
Female	26 (40)	12 (0–36)		76.4 (63.8–95.3)		34 (17.5–44.7)	
Not specified	1 (2)						
Age group							
Less than 45	1 (2)	Missing	0.07	48	0.049	16	0.28
45–54	7 (11)	7.5 (0–43.5)		63.3 (37.4–86)		26 (13–31)	
55–64	18 (28)	12 (7.5–23)		69.8 (59.1–92.8)		35.5 (20.5–43.8)	
65–75	27 (42)	21 (6–39)		84.4 (69.5–100.7)		35 (24–48)	
Over 75	12 (18)	1.5 (0–15.8)		65.4 (46.8–90.9)		23 (10–44.4)	
Years since MM diagnosis							
Less than 1	9 (14)	19.5 (1.5–33.3)	0.92	75 (58.1–89.4)	0.75	37 (16–44.7)	0.57
1 to 5	28 (43)	15 (6–34.5)		71.5 (55.7–94)		31 (14.25–39.8)	
6 to 10	14 (22)	15 (3–36)		74.9 (65.8–103.2)		35 (24–49.3)	
Over 10	8 (12)	12 (0–58.6)		80.1 (66.1–95)		34 (23–43)	
Not specified	6 (9)						
On myeloma treatment							
Yes	43 (66)	12 (3–21)	0.16	72 (59.1–92.4)	0.37	26.5 (16.8–40.3)	0.34
No	21 (32)	26.5 (6.8–51)		77.7 (63.5–98)		35 (19.5–48.5)	
Not specified	1 (2)						
Line of myeloma treatment							
None	7 (11)	25.5 (0–42.3)	0.43	78.4 (65.3–104)	0.72	39 (23–50)	0.30
Radiotherapy alone	3 (5)	33		66		17	
1st	17 (26)	20 (7.5–29)		68 (57.2–91.6)		34 (24–39.5)	
2nd	9 (14)	12 (3–28)		76.5 (46.6–97.8)		31 (11.5–50)	
3rd or beyond	17 (26)	9 (0–15)		72.4 (54.4–91.4)		23.5 (14.5–37.8)	
Not specified	12 (18)						

p-values are displayed when comparing between independent groups

**Table 2:** Perceived barriers preventing patients from improving their level of physical activity

Perceived barriers preventing patients from improving their PA*	Frequency (%)
None	16 (25)
Weakness	28 (43)
Fatigue	26 (40)
Pain	17 (26)
Neuropathy from myeloma treatment	13 (20)
Known myeloma bone disease	10 (15)
Known arthritis	6 (9)
Previous surgery limiting mobility	5 (8)
Others	2 (3)

*Patients could choose more than one option. 71% of those who indicated barriers to PA reported that a combination of these factors, rather than a single factor, prevented them from improving their exercise levels

Fifty-nine percent would like to receive PA advice from a HCP. The three most selected options to receive such advice were written leaflets (29%), a face-to-face session with a HCP (28%) and verbal advice in clinic consultation (23%). Other chosen methods include a telephone call from a HCP (14%), mobile application (12%) and internet website (11%). None of them chose group seminar.

Our respondents had a median GLTEQ score of 15 (range 0–66). According to their scores, 25% were deemed physically active, 17% moderately active and 39% inactive. Thirteen (20%) did not specify their PA level. The median FACT-G score was 73.1 (range 31.2–108, mean 75.1 ± 21.1), and the median FACIT-F score of 32.5 (range 3–52, mean 30.3 ± 14.6). GLTEQ, FACT-G and FACIT-F scores were not significantly different between males and females, and between those on or off myeloma treatment. Nor did these scores correlate with years since myeloma diagnosis, or with numbers of prior treatment lines (Table 1). These scores did not differ significantly between age groups, apart from FACT-G, where the scores in 65–75 were higher than those in 45–54 and in > 75 s (p = 0.049). Using a cut off FACIT-F score of 34[13], 49% of the patients were classified as clinically fatigued. These clinically fatigued patients had a lower median GLTEQ score than those who were not (p < 0.001).

**Fig. 1:**
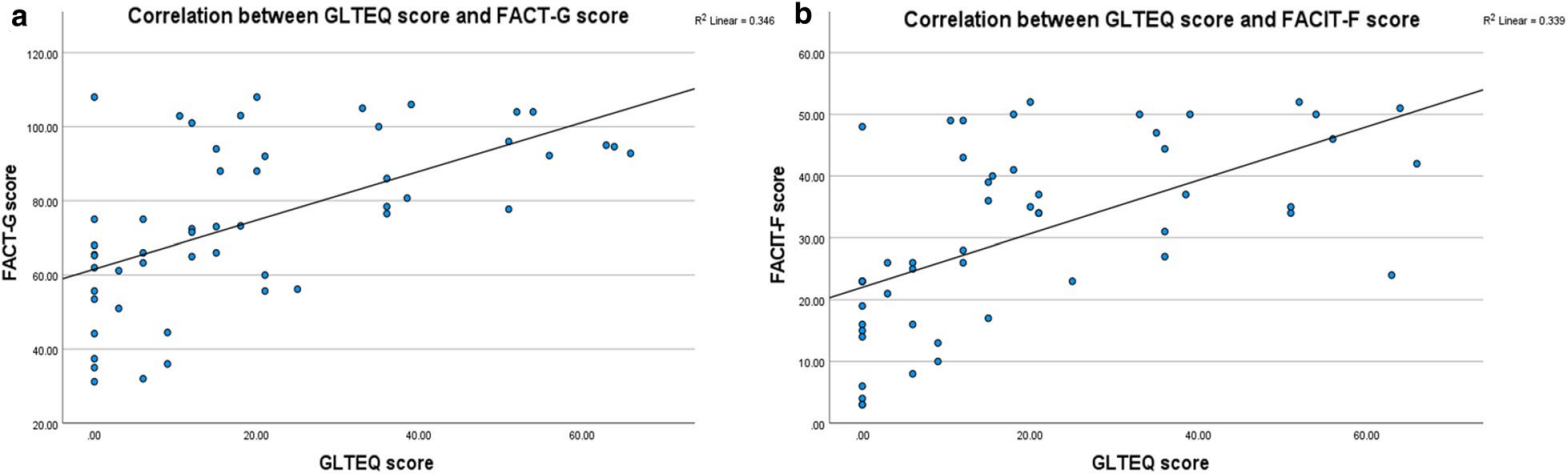
**a** Scatterplot showing a positive correlation between the GLTEQ score and the FACT-G score (Spearman’s correlation coefficient 0.62, n = 52, p < 0.001), suggesting that a higher physical activity level was associated with better quality of life. **b** Similarly, there was a positive correlation between the GLTEQ score and the FACIT-F score (Spearman’s correlation coefficient 0.67, n = 52, p < 0.001), suggesting that a higher physical activity level was associated with a lower level of fatigue

Finally, there was a significant positive correlation between GLTEQ and FACT-G scores (Spearman’s correlation coefficient = 0.62, n = 52, p < 0.001), and also between GLTEQ and FACIT-F scores (Spearman’s correlation coefficient = 0.67, n = 52, p < 0.001) (Fig. 1).

### Discussion

One key finding is the low PA level in our cohort of real-world MM patients. Although a different self-reported measure was used, our figure of 25% is much lower than the 67% of U.K. general population who were considered active (ages 75–84—53%, age 85+—31% active) [14]. These low PA levels are hardly surprising; in addition to the fear of injury, related to their bone disease, MM patients suffer with a considerable symptom burden, related to their older age and co-morbidities, as well as treatment toxicities. Barriers to exercise are usually multifactorial, but neuropathy was featured in 20% of our participants, alongside more common cancer-related symptoms such as fatigue and pain. Peripheral neuropathy is a common and often painful side effect of some of the commonest agents used to treat myeloma, including bortezomib and thalidomide. Similar to our findings, Craike et al. [15] reported that most barriers were related to MM symptoms and treatment toxicities. These factors inevitably have a negative impact on QoL, which is reflected by the lower mean FACT-G score in our cohort compared to that of the general population (75.1 versus 80.1) [16]. Interestingly, however, there was no meaningful difference in GLTEQ and FACT-G scores between those who were on and off myeloma treatment in this study. Furthermore, those who were 65–75 had higher FACT-G scores than two other age groups. These observations may be explained by the small sample size, and the multiple confounding physical and psychosocial factors that could influence these scores.

It is also noteworthy that while most patients had low exercise levels, the majority would like to improve this. This suggests an awareness of the possible benefits of exercise, and an unmet survivorship need. Over half of patients wished to receive PA advice from a HCP, suggesting that one of the barriers to exercising may be lack of confidence to do so safely. This echoes themes reported in existing literatures [6, 7], where patients were more confident to exercise under supervision by a physiotherapist experienced in working in myeloma, and reflects the need for development of specialist physiotherapy workforce for this complex patient group. Oncology HCPs have also reported lack of clear guidelines and not being the right person to give PA and lifestyle advice [17].

Fatigue is a ubiquitous problem in cancer patients. In MM, it is compounded by the use of steroids in almost every treatment line, bone pain and fractures, and the autonomic neuropathy that accompanies many anti-myeloma drugs. Our patients had a lower mean FACIT-F score than that of the general population (32.5 versus 43.6) [16]. Almost half (49%) were classified as clinically fatigued (FACIT-F score < 34) and unsurprisingly, they had significantly lower GLTEQ scores than those who were not fatigued (p < 0.001). Patients with fatigue are often advised, or believe it is best, to rest to conserve energy. Excessive rest results in loss of functional capacity through deconditioning. Deconditioning and increasing effort to undertake daily activities, further exacerbates fatigue. Disrupting this cycle through exercise is key to its management [18]. Our study demonstrated an association between higher PA levels and lower fatigue. The well documented beneficial effects of exercise on cancer-related fatigue, and on bone health, further highlights this unmet need in myeloma patients.

We observed that higher PA levels were associated with better QoL, in accordance with previous studies [3–5]. Although current study design does not allow a causal relationship to be concluded, existing evidence has shown that PA improves health related QoL [19]. Interestingly, for those who were physically active (GLTEQ score > 24, n = 16), PA levels did not correlate with QoL (Spearman’s correlation coefficient 0.21, p = 0.43), nor did it with lower fatigue levels (Spearman’s correlation coefficient 0.25, p = 0.35). This could indicate a possible association threshold with PA and could potentially be used as a target activity level for patients to achieve.

MM patients have lifelong follow up in outpatient clinics, where emphasis is often placed on myeloma biochemical markers rather than patients’ survivorship needs. Our study results highlight an unmet need to develop a more holistic care model, in which PA is assessed and promoted appropriately, with professional support from physiotherapists as appropriate. It is also important to identify and address barriers to PA proactively, such as through pain and neuropathy management. In line with MM supportive care guideline [20], regular PA should be encouraged from diagnosis to ensure that prehabilitation and rehabilitation are an integral part of every treatment line. To achieve this, it is key that both the patient and the clinical team are educated on the individual benefits and risks of exercise. To promote survivorship and self-management, our team designed and runs a pilot multidisciplinary team (MDT) MM clinic involving a doctor, a nurse specialist and a physiotherapist, with wider MDT support if needed. Tailored exercise advice is given in each consultation and patients are encouraged to set achievable goals. They are signposted to various survivorship tools to help maintain physical and psychosocial wellbeing. Data on patient reported outcomes and patient experience are being collected to evaluate this alternative care model (REC Ref: 19/NS/0105).

A more formal way to promote PA is to deliver supervised exercise programmes, which, when administered by specialist personnel with appropriate screening procedures, have been shown to be safe and feasible [6]. Specialists should be involved so that MM related symptoms and treatment toxicities are taken into account in the prescription of individualised programmes. With limited literatures available, more high-quality, large randomised studies are needed to evaluate the impact of PA on different health parameters of MM patients. Research protocols such as PREeMPT [21] and PERCEPT studies [22], which look at prehabilitation before ASCT, will provide information to support for the use of exercise intervention in the future. Although such programmes may be a positive way to influence PA, only 28% of our patients preferred to receive PA advice in a face-to-face session. This suggests that formal exercise classes may not suit everyone, and clinicians need to devise flexible ways to promote PA depending on patients’ preference, especially when digital healthcare technologies are becoming increasingly popular.

## Limitations

- Results are limited by the cross-sectional nature of this single-site study, and by small sample size.
- Self-report data is subjected to bias and limitations such as introspective ability and interpretation of questions.
- Associations demonstrated in the study have no causal relationship due to its design.

## Supplementary Information

**Additional file 1.** Physical activity survey.

## Data Availability

The datasets used and/or analysed during the current study are available from the corresponding author on reasonable request.
