# Supplementary material for: Multiple myeloma and physical activity
Source: BMC Res Notes. 2021 May 7;14:171. doi: 10.1186/s13104-021-05591-y (PMC8103584; doi:10.1186/s13104-021-05591-y)
Supplement: Supplementary file 1 — Additional file 1. Physical activity survey. [file 13104_2021_5591_MOESM1_ESM.pdf]

Thank you very much for taking part in this short survey about physical activity in myeloma patients. You will only have to fill in this form once when offered. Please circle the most appropriate answers and kindly return the completed questionnaire to reception.

1. Please state your age group:

<45	45-54	55-64	65-75	>75

2. Gender:

Female	Male

3. Are you currently receiving any myeloma treatment?

Yes	No – on active surveillance

4. How many years ago was your myeloma diagnosed? _________

5. What line of myeloma treatment are you currently on, or most recently received? (including different types of chemotherapy and radiotherapy)

I have not had any treatment	First line	Second line
Third line or beyond	Only radiotherapy	

6. You would like to increase your level of physical activity:

Strongly disagree	Disagree	Neither agree nor disagree	Agree	Strongly agree

7. Is there anything that may prevent you from increasing your level of physical activity? (can circle more than one box)

No	Yes – Pain
Yes – Fatigue	Yes – Weakness
Yes – Previous surgery limiting mobility	Yes – Known bone disease associated with myeloma
Yes – Known arthritis	Yes – Neuropathy from myeloma treatment
Other (please specify):	

8. Would you like advice from healthcare professionals regarding physical activity?

Yes	No (Please turn overleaf)

9. How would you like to receive the information? (can circle more than one box)

Verbal advice in clinic appointment	Printed leaflet	Internet website
Mobile app	Telephone call from a healthcare professional	Face to face session, separate from clinic
Group seminar	Other (please specify):	

Please turn over
